# Osteoclast Heterogeneity in Osteoarthritis: From Single-Cell Microenvironments to Program-Specific Therapeutic Opportunities

**DOI:** 10.3390/ijms27114838

**Published:** 2026-05-27

**Authors:** Tingxuan Tang, Peidong Zhang, Zhiqiang Lin, Zhao-Hui Tang, Deng Chen

**Affiliations:** 1 Department of Orthopedics, Tongji Hospital, Tongji Medical College, Huazhong University of Science and Technology, Wuhan 430030, China; tangtingxuan@hust.edu.cn; 2 Department of Trauma Surgery, Emergency Surgery & Surgical Critical, Tongji Trauma Center, Tongji Hospital, Tongji Medical College, Huazhong University of Science and Technology, Wuhan 430030, China; peidongzhang@hust.edu.cn (P.Z.); linzhiqiang@hust.edu.cn (Z.L.); tangzh@tjh.tjmu.edu.cn (Z.-H.T.)

**Keywords:** osteoarthritis, osteoclast-lineage heterogeneity, subchondral bone remodeling, single-cell omics, osteochondral microenvironment, precision therapeutics

## Abstract

Global disability from osteoarthritis (OA) remains a staggering burden, yet disease-modifying pharmacological therapies remain limited. Current evidence supports OA as a whole-joint disease in which subchondral bone remodeling acts as an early and active driver of structural progression and pain. Osteoclasts are key regulators of this process, but clinical trials targeting osteoclast-dependent remodeling have yielded inconsistent results. This translational gap suggests that the traditional view of osteoclasts as a homogeneous, terminally differentiated resorptive population is no longer sufficient. Latest breakthroughs in single-cell and spatial multi-omics have begun to redefine osteoclast biology by revealing heterogeneity across precursor origin, lineage state, functional output, and niche-specific adaptation. In OA, these studies have clarified the osteoclastogenic microenvironment more clearly than the terminal taxonomy of mature osteoclast subsets, thereby shifting the field toward a state-spectrum framework. In this review, we synthesize recent high-resolution evidence to examine how osteoclast-lineage heterogeneity is organized across disease stages and osteochondral microenvironments, and how distinct osteoclast-lineage programs contribute to subchondral remodeling, angiogenic coupling, interface instability, and pain-related pathology. We further discuss how this framework may inform patient stratification, mechanism-matched intervention, and the development of program-specific therapies in OA.

## 1. Introduction

Global disability from osteoarthritis (OA) remains a staggering burden, yet a true disease-modifying pharmacological therapy continues to evade us [1,2]. The field has long since outgrown the primitive, cartilage-centric view of “wear-and-tear.” We now recognize OA as a total-joint failure where cartilage, subchondral bone, synovium, and neurovascular elements deteriorate in an interconnected, pathological spiral [3,4]. In this landscape, subchondral bone remodeling has emerged as a primary driver of both structural decay and the clinical experience of pain—particularly through its roles in bone marrow lesions (BMLs) and aberrant osteochondral crosstalk [3,5]. At the center of this process is the osteoclast. Far from being mere “scavengers” of late-stage damage, these cells act as early orchestrators that shape the multicompartmental environment of the osteochondral unit [6,7,8].

If osteoclasts are indeed the chief architects of this remodeling, then antiresorptive strategies should, in theory, stop OA in its tracks. Preclinical data often promise exactly that, showing clear benefits in early-stage bone loss and pain signaling. Clinical reality, however, tells a different story. Trials of bisphosphonates for knee OA have been, frankly, underwhelming or inconsistent; only a fraction of studies show even modest short-term relief [9,10]. This translational gap suggests a fundamental oversight. The question is not simply whether osteoclasts matter, but rather which specific osteoclast program is being targeted, in which patient, and at what stage. Our long-standing assumption that the osteoclast is a single, monolithic pathogenic effector is simply too reductive to capture the heterogeneity of OA itself [9,10].

This traditional “one-size-fits-all” model is becoming impossible to sustain. Recent high-resolution single-cell and spatial multi-omics reveal that the osteoclast lineage is not a terminal, fixed phenotype. Instead, it is a mosaic [3,11,12,13]. We now see a diversity that spans from the very origin of the cell—distinct precursor pools and immune priming—to its functional outputs, which can be resorptive, angiogenic, or even neurotrophic [3,7,8,11,14]. This heterogeneity provides a much-needed explanation for the clinical puzzles we face: why bone-targeted drugs work only in specific windows, and why structural damage, neurovascular invasion, and pain so often appear uncoupled in different patients [3,7,8].

Against this backdrop, this review re-examines OA through the lens of osteoclast diversity. Our core premise is that OA-relevant biology is better defined as a dynamic “state spectrum” rather than a static list of cell subsets. By integrating evidence from single-cell atlases and spatial profiling, we aim to map the cellular ecosystems that give rise to these specific programs. Ultimately, we address three critical questions: How is this lineage organized in the OA joint? What do the latest high-resolution data actually tell us? And how can we translate this complexity into program-specific, precision therapies?

## 2. From Osteoclast Heterogeneity to a State-Spectrum Framework: Concise Conceptual Background

Osteoclasts are classically defined as multinucleated, TRAP-positive cells specialized for bone resorption [15]. That definition remains valid, but it does not capture the full biological range now attributed to the osteoclast lineage. Evidence from single-cell and spatial studies indicates that osteoclast biology cannot be reduced to one invariant terminal phenotype; instead, heterogeneity emerges across precursor source, differentiation trajectory, microenvironmental instruction, and effector output [12,13,16]. For OA, this conceptual shift is not a semantic refinement. It provides the framework required to interpret why osteoclast-lineage cells may contribute to distinct structural, vascular, interface-related, and pain-relevant disease programs rather than acting as a uniform osteolytic population.

### 2.1. Sources of Heterogeneity Across the Osteoclast Lineage

A major part of osteoclast heterogeneity is established before multinucleated resorbing cells appear. Osteoclasts arise from multiple precursor reservoirs rather than from one interchangeable precursor pool, including circulating monocytes, marrow-resident precursors, and local macrophage-related myeloid populations [15,17,18]. These inputs are not equivalent. They differ in migratory behavior, inflammatory responsiveness, tissue residence, and readiness to enter osteoclastogenic programs. As a result, osteoclast-lineage diversity is partly determined by which precursor populations are recruited and licensed within a given tissue context. This point is particularly relevant to OA, where mechanical stress, marrow remodeling, vascular change, and inflammatory signaling are unlikely to prime all precursor pools in the same way. Osteoclasts supply may also be supplemented by recycling mechanisms, as osteomorph biology suggests that mature osteoclast-lineage biomass can be retained and re-enter the remodeling process under renewed osteoclastogenic drive [19,20].

### 2.2. Recurrent Osteoclast-Lineage Programs Relevant to OA

While a definitive taxonomy of the osteoclast lineage remains out of reach, the literature now points toward several recurring “programs” that drive OA pathology [12]. These are not mere descriptive labels; they represent distinct biological modes through which the lineage participates in the disease.

At the foundation is the canonical resorbing state, the well-characterized machine optimized for acidification, proteolysis, and the excavation of mineralized surfaces [21]. However, the lineage is not confined to conventional bone surfaces. At the mineralized cartilage junction, we find “interface-adapted” or chondroclast-like cells that must navigate a unique biochemical and physical landscape. Perhaps even more critical in the early stages are the pre-osteoclasts. These early-lineage cells often exert their most profound influence—through potent paracrine signaling and niche remodeling—long before they ever reach full multinucleation or maximal resorptive power [13,16,22]. In inflammatory endotypes, the lineage takes on a different character altogether: an “inflammatory” state licensed by cytokine-rich or danger-signal-rich environments [13,22]. Finally, the emerging concept of “osteomorphs” introduces the possibility of a recycling mechanism, where osteoclast biomass is redistributed and reactivated rather than simply lost to apoptosis [19,20]. For the clinician and researcher alike, the importance of these programs lies in their functional diversity—each one offering a different path through which the osteoclast lineage shapes the OA joint.

### 2.3. A Dynamic State Spectrum Is More Useful than a Static Subsets Catalog

**Figure 1:**
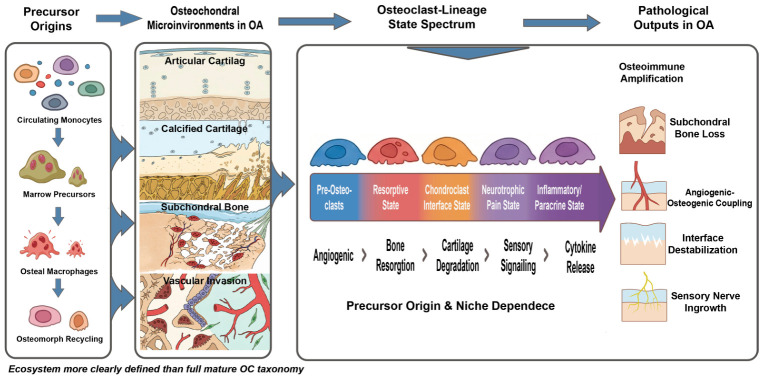
Osteoclast-lineage heterogeneity in osteoarthritis is organized as a stage- and niche-dependent state spectrum. Schematic overview of how unequal precursor inputs, osteochondral microenvironments, and disease stage shape distinct osteoclast-lineage programs in OA. Current high-resolution studies most clearly define the osteoclastogenic ecosystem, including subchondral vascular-stromal niches, bone marrow lesion-associated myeloid states, and osteochondral coupling networks. These niche-dependent programs contribute to subchondral bone loss and instability, angiogenic–osteogenic coupling, osteochondral interface disruption, pain-related sensory innervation, and disease amplification.

These observations support a framework in which osteoclast heterogeneity is interpreted as a dynamic state spectrum rather than as a fixed catalog of stable subsets [13,16]. A static subsets model assumes that osteoclasts populations can be cleanly partitioned into discrete and persistent cell types. Current evidence argues for a more fluid view. Osteoclast-lineage cells appear to move across states defined by precursor origin, tissue location, local instruction, inflammatory licensing, and functional output. This framework is particularly useful in OA because the dominant osteoclast-lineage programs is unlikely to remain constant across disease stages, lesion types, and osteochondral microdomains. It is also more compatible with current high-resolution evidence, which often resolves precursor logic and local niche organization more clearly than mature multinucleated osteoclasts states themselves. This state-spectrum view provides the conceptual bridge between general osteoclast biology and OA-specific disease mechanisms; a schematic overview is shown in Figure 1.

## 3. Single-Cell Studies Define the Osteoclastogenic Ecosystem in OA

Latest single-cell multi-omics and spatially resolved profiling have transformed the OA field, but their strongest contribution has been to define the osteoclastogenic ecosystem rather than to generate a complete atlas of mature OA osteoclast subsets [23,24,25,26,27,28,29]. Current datasets consistently show that osteoclast-lineage behavior in OA is embedded within a structured osteochondral microenvironment, yet direct high-resolution characterization of mature multinucleated osteoclasts remains incomplete. The field has therefore moved furthest in clarifying where and under what conditions osteoclast-lineage programs arise, rather than in fully resolving every terminal osteoclast states in native OA tissue.

### 3.1. Subchondral Bone Atlases Identify Osteoclast-Lineage Activation

The clearest direct evidence comes from single-cell analysis of subchondral bone from OA patients which demonstrates that osteoclast-lineage activity is nested within a highly organized vascular-stromal-osteogenic niche [23,24]. In the RMD Open study by Hu and colleagues, OA subchondral bone was resolved into multiple cell populations, with pronounced endothelial and osteoblast-lineage heterogeneity [23]. The endothelial compartment included populations enriched for inflammatory and exosome-related programs as well as populations more tightly linked to vascular behavior and angiogenesis; the osteoblast lineage was similarly partitioned into states associated with vascularization, matrix production, and mineralization [23]. Even though mature osteoclasts themselves were not deeply captured, the implication is direct: OA subchondral remodeling occurs within a cellular environment that is already pre-structured to regulate osteoclast-lineage commitment [23].

This insight changes the interpretive frame of osteoclast biology in OA. Instead of asking only whether Osteoclast number or activity is increased, one can now ask which combinations of endothelial, stromal, and osteogenic states create permissive or amplifying conditions for osteoclast-lineage activation at specific osteochondral sites [23,24,25]. In other words, single-cell studies have shifted the field from a purely cell-autonomous model of osteoclast activation toward a microenvironmental model in which local tissue organization is itself a determinant of osteoclast heterogeneity.

### 3.2. Bone Marrow Lesion Studies Move the Field Upstream to Precursor Heterogeneity

**Figure 2:**
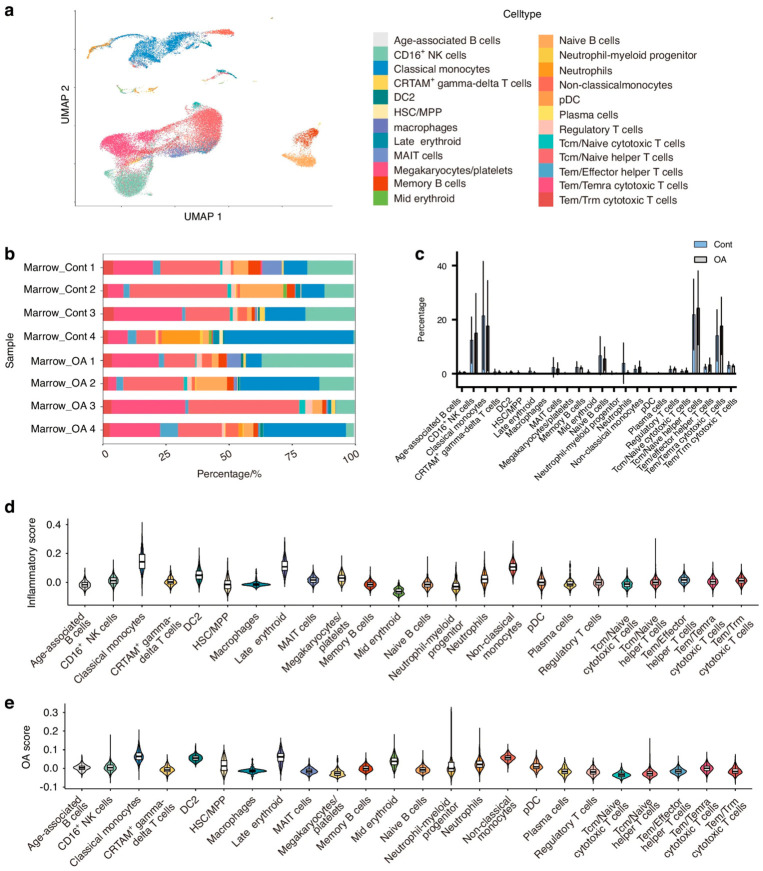
Single-cell immune landscape of OA bone marrow lesions highlighting upstream myeloid heterogeneity. Reproduced with permission from Lou et al., *Bone Research* 2025;13:94. DOI:10.1038/s41413-025-00467-4 [27]. (**a**) UMAP visualization of integrated bone marrow cells from non-BML control samples and BML OA samples. (**b**) Relative donor contribution to each cluster across individual samples. (**c**) Proportions of individual cell clusters in the control and OA groups, illustrating between-sample variability in cluster abundance. (**d**) Inflammation signature scores across clusters. (**e**) OA-related signature scores across clusters. In the context of this review, the main relevance of this figure is not that every cell population shows a uniformly large abundance shift between control and OA samples, but that OA bone marrow lesions represent biologically active and heterogeneous immune niches, with disease-related inflammatory and OA-associated transcriptional activation present at the precursor level. This supports the view that osteoclast-lineage heterogeneity in OA is shaped in part upstream, through marrow immune and myeloid states.

BMLs provide a second major line of direct evidence and place osteoclast heterogeneity in a precursor-centered context [27]. Single-cell analysis of OA BMLs has revealed an immune landscape marked by inflammatory activation and cellular senescence, with non-classical monocytes showing elevated inflammatory scores, OA-associated transcriptional features, and senescence-related signatures [27]. These cells also engage in TNF-centered crosstalk with cartilage-associated states, indicating that BMLs are biologically active lesions rather than passive imaging correlates. This precursor-centered view is further supported by single-cell profiling of OA bone marrow lesions, which highlights a heterogeneous immune niche with substantial between-sample variability but clear disease-related inflammatory and OA-associated transcriptional activation at the precursor level, as shown in Figure 2 [27].

For osteoclast biology, the significance of these findings lies in their timing within the lineage hierarchy. They suggest that a substantial component of OA-associated osteoclast heterogeneity is established before terminal clastic differentiation, at the level of myeloid precursor pools that differ in inflammatory tone, osteoclastogenic competence, and niche-remodeling capacity [27]. This makes BMLs especially informative for understanding bone-active and pain-associated OA phenotypes, because they link marrow pathology, inflammatory precursor asymmetry, and osteochondral crosstalk within the same microdomain [14,27].

### 3.3. Osteochondral Composite Atlases Place Osteoclast-Lineage Programs Within a Broader Coupling Network

A complementary set of direct insights comes from single-cell studies of osteochondral composite tissues, which extend the analysis beyond subchondral bone alone and place osteoclast-lineage behavior within a wider network of angiogenesis, osteogenesis, and interface destabilization [29]. In the recent study by Liu and colleagues, single-cell transcriptomics identified angiogenic chondrocyte populations and pathogenic osteoblast states linked to abnormal subchondral remodeling [29]. These findings do not define mature osteoclast subsets directly, but they substantially strengthen the interpretation that osteoclast-lineage programs in OA are coupled to vascular invasion, abnormal mineralization, and cartilage-bone communication rather than to resorption alone [29]. Experimental evidence has shown that PDGF-BB derived from mononuclear pre-osteoclasts can drive pathological subchondral angiogenesis and promote H-type vessel formation [30]. Osteochondral single-cell atlases make such findings more intelligible at the tissue level, because they reveal neighboring chondrocyte and osteoblast states that could participate in the same angiogenic-osteogenic circuit [29,30]. These data suggest that osteoclast heterogeneity in OA is not limited to variation in resorptive output; it also reflects the extent to which osteoclast-lineage cells are embedded in coupling programs that reshape the osteochondral unit. This boundary should be stated explicitly.

### 3.4. Unresolved Problems at Single-Cell Resolution

Despite these advances, current OA datasets define the osteoclastogenic microenvironment more clearly than mature multinucleated osteoclasts states themselves [23,24,25,26,27,29]. Mature osteoclasts are relatively rare in human OA samples, difficult to preserve during tissue dissociation, and systematically underrepresented in studies of mineralized tissues [23,24,25,26]. Spatial transcriptomics preserves anatomical context and partly mitigates this limitation, but it does not yet fully overcome the challenge of assigning mature osteoclasts states with both high spatial precision and full transcriptional depth [25,26].

This boundary should be stated explicitly rather than treated as a weakness. At present, the strongest conclusion supported by high-resolution studies is that OA contains distinct microenvironments that license different osteoclast-lineage programs. What remains incomplete is the direct terminal taxonomy of mature OA osteoclast subsets. That distinction is methodologically important and conceptually useful, because it allows the osteoclast heterogeneity framework to remain biologically grounded without overstating the maturity of the current atlas.

## 4. Osteoclast Heterogeneity in OA: A Unified Disease Model

Defining osteoclast heterogeneity is central to a mechanistic understanding of OA and has direct implications for therapeutic stratification [31]. In OA, osteoclast heterogeneity is more appropriately viewed as a dynamic continuum of lineage states shaped by precursor origin, differentiation status, spatial niche, and functional output, rather than as a fixed catalog of discrete subsets [32,33]. This perspective places osteoclast pool within the broader osteochondral microenvironment, where they participate in subchondral remodeling, angiogenesis, sensory innervation, interface disruption, and inflammatory amplification across the course of disease [14,23,27,28,32,33].

### 4.1. Precursor and Lineage Heterogeneity Underlies the Osteoclast Pool in OA

A substantial component of osteoclast heterogeneity in OA is established upstream, before multinucleated resorbing cells emerge. Circulating monocytes constitute one major source of osteoclast precursors, but their contribution is not uniform. Exposure to damage-associated molecular patterns, cytokines, and lipid mediators modifies their osteoclastogenic competence before tissue entry, while chemokine gradients determine which myeloid subsets are preferentially recruited into synovium and subchondral marrow [34]. Within this framework, the CCL2-CCR2 axis appears to function as a critical gatekeeper, particularly in synovitis-associated and post-traumatic OA, where inflammatory activation enhances myeloid influx and shifts the local milieu toward osteoclasts genesis [35].

This heterogeneity becomes more evident in BMLs. Single-cell analyses of OA BMLs have identified an immune landscape marked by inflammatory activation and senescence, with non-classical monocytes showing elevated inflammatory scores, OA-associated transcriptional features, and senescence-related signatures, together with TNF-centered crosstalk with cartilage-associated cell states [27]. These observations indicate that heterogeneity within the osteoclast lineage in OA begins with nonequivalent upstream myeloid populations that differ in osteoclastogenic potential, inflammatory tone, and niche-remodeling capacity.

Local precursor pools provide an additional layer of complexity. Single-cell profiling of subchondral bone derived from patients with OA have shown that osteoclasts genesis takes place within a structured cellular ecosystem composed of immune-myeloid, endothelial, mesenchymal, and osteoblast-lineage compartments [23,24,32]. Although mature osteoclasts remain poorly represented in currently available human OA scRNA-seq datasets, the overall architecture of these datasets strongly supports the concept that osteoclast-lineage commitment is instructed locally by vascular and stromal niches rather than by circulating precursors alone [23,24,32]. Osteal macrophages may contribute to this process by regulating osteoblast and stromal behavior and by serving as a locally available myeloid reservoir that can be mobilized when osteoclastogenic cues intensify [36].

Lineage plasticity adds a further dimension. Intravital imaging and single-cell studies have shown that multinucleated Osteoclasts may undergo fission to generate smaller osteomorph-like progeny that retain lineage identity and can later re-fuse into functional resorbing cells [19,20]. In OA, such recycling could sustain intermittent and spatially shifting bursts of osteoclast activity without repeated de novo differentiation [19,20].

### 4.2. Temporal Heterogeneity Across Disease Progression

OA-associated osteoclast heterogeneity also has a pronounced temporal dimension. In early disease, osteoclast-lineage cells are biased toward a high-turnover, remodeling-initiating state. Increased Osteoclast number and activity in subchondral bone accelerate bone resorption, enlarge marrow cavities, reduce mineral density, and disrupt trabecular and plate architecture before cartilage degeneration becomes the dominant histological feature [14,23,28,37,38]. Under these conditions, osteoclast act as an early structural driver of osteochondral disequilibrium.

As OA progresses, osteoclast function becomes increasingly embedded in uncoupled remodeling, sclerosis, tidemark advancement, vascular channel formation, and osteophyte maturation [36,39,40,41]. The dominant osteoclast program in OA is therefore not static. In this sense, OA does not sustain a single osteoclast program across progression. Early resorptive activity gives way to later, more spatially constrained states that are integrated with sclerosis, coupling, and structural reorganization [19,20].

### 4.3. Spatial Heterogeneity Within the Osteochondral Unit

A further defining feature of OA-related osteoclast heterogeneity is its dependence on spatial context. The osteochondral unit is composed of multiple microdomains, including the subchondral plate, trabecular marrow-facing surfaces, BMLs, vascular channels, and the tidemark-calcified cartilage interface [23,27,42]. These regions differ in biomechanical loading, vascularization, inflammatory tone, matrix composition, and permeability. Osteoclast-lineage programs are therefore shaped by location as much as by lineage history.

Single-cell studies of human OA subchondral bone provide direct evidence for marked niche heterogeneity. These datasets have identified precursor endothelial populations enriched for inflammatory and exosome-related programs, angiogenic endothelial cells, and three osteoblast subtypes linked to vascularization, matrix production, and matrix mineralization [23,24,32]. A complementary single-cell atlas of osteochondral tissues has further identified angiogenic chondrocyte populations and pathogenic osteoblast states associated with H-type vessel formation and aberrant subchondral remodeling [29]. These findings suggest that osteoclast heterogeneity in OA cannot be interpreted independently of the surrounding vascular-stromal environment.

The BMLs compartment contributes another spatially distinct niche. scRNA-seq analyses have shown enrichment of inflammatory and senescent monocyte populations within BMLs, together with direct signaling interactions between marrow-derived immune cells and cartilage-associated cell states [27]. This supports the view that trabecular marrow and BMLs represent privileged osteoclastogenic environments characterized by inflammatory activation, cellular stress, and extensive marrow-cartilage communication. From this perspective, spatial heterogeneity in OA is best understood as niche dependence rather than as simple anatomical variation.

### 4.4. Resorptive States and Angiogenic-Osteogenic Coupling

The best-characterized osteoclast state in OA is the resorptive osteoclasts localized to focal subchondral remodeling fronts enriched in osteoclastogenic signals [14,23,28]. These cells are expected to exhibit high expression of acidification machinery, lysosomal pathways, cytoskeletal polarization modules, and matrix-degrading enzymes. Their activity weakens the mechanical integrity of the osteochondral unit by increasing plate porosity and trabecular instability, thereby altering load transfer and rendering cartilage more susceptible to damage [14,23,28].

OA-related osteoclast heterogeneity, however, extends beyond canonical bone resorption. Mononuclear pre-Osteoclasts and early lineage intermediates may adopt paracrine states with limited resorptive function. A representative example is a PDGF-BB-producing pre-osteoclast state that drives pathological subchondral angiogenesis and promotes formation of CD31^high^Emcn^high^ H-type vessels [30]. These vascular changes recruit osteoprogenitors and facilitate aberrant bone formation, placing osteoclast-lineage cells within a broader angiogenic-osteogenic circuit [30]. This view is further supported by single-cell osteochondral studies showing that Angptl7^+^ chondrocytes stimulate subchondral angiogenesis via FGF2-FGFR2 signaling, whereas Sparc^+^ osteoblasts drive pathological remodeling and defective mineralization [29]. Within OA, one major form of osteoclast heterogeneity therefore lies in the transition from a predominantly resorptive states to one coupled to angiogenesis and maladaptive osteogenesis.

### 4.5. Osteochondral Interface Programs and Pain-Coupled States

At the osteochondral interface, OC-lineage cells may acquire a chondroclast-like program adapted to calcified cartilage degradation, tidemark advancement, vascular channel extension, and increasing osteochondral connectivity [43,44,45,46]. This program is not adequately described as a simple relocation of conventional bone-resorbing Osteoclasts. It is more consistent with an interface-adapted lineage state shaped by altered matrix composition, increased permeability, and vascular-stromal support within the calcified cartilage and tidemark region [43,44,45].

Pain represents a second, and clinically distinct, output of osteoclast-lineage heterogeneity. A distinct functional state is linked to pain. Osteoclast-derived netrin-1 promotes aberrant sensory nerve in growth in subchondral bone and contributes to pain behavior, whereas interference with Netrin-1-DCC signaling or suppression of osteoclasts activity alleviates nociceptive responses [14]. In this setting, the defining pathological output is neural recruitment rather than the absolute magnitude of matrix resorption. This pain-associated osteoclast state is particularly relevant to BML-associated OA phenotypes, in which enhanced osteoclasts activity, marrow pathology, and nociceptive sensitization frequently coexist [14,27].

### 4.6. Osteoimmune and Paracrine Heterogeneity

OA-related osteoclast heterogeneity is embedded in osteoimmune and paracrine crosstalk. Inflammatory OC-lineage states are licensed by NF-κB-, TNF-, and IL-1-related programs, DAMP sensing, and macrophage activation circuits, and are most evident in synovitis-dominant OA and inflammatory flares after joint injury [47,48]. Under these conditions, osteoclasts coexist with activated myeloid populations and inflammatory stromal cells, which together sustain osteoclastogenic tone and tissue-destructive signaling [23,47,48].

Lineage-derived biological products represent another relevant dimension. OC-derived apoptotic bodies accumulate in OA subchondral bone and promote MSC osteogenesis through RANKL reverse signaling, thereby contributing to aberrant remodeling and structural progression [49]. This expands the concept of osteoclast heterogeneity beyond the properties of living cells themselves and points to a broader spectrum of osteoclast-lineage outputs. Secretome-related mechanisms, including extracellular vesicles and other paracrine mediators, are likely to represent an additional layer of OA-related osteoclast heterogeneity, although they remain less well defined.

Current single-cell studies in OA resolve the osteoclastogenic ecosystem more clearly than mature osteoclast subsets themselves [24,27,29]. Even so, the available evidence supports a model in which OA-associated osteoclast heterogeneity comprises distinct precursor reservoirs, early high-resorption states, angiogenic and osteogenic-coupled pre-OC states, interface-adapted chondroclast-like states, pain-coupled neurotrophic states, and inflammatory or paracrine disease-amplifying states. This framework also helps explain why antiresorptive interventions may yield variable effects across disease stages and OA phenotypes [14,24,32,33].

## 5. Osteoclast Heterogeneity in OA: Translational Implications for Clinical Therapy

While the osteoclast is undoubtedly the lead actor in bone resorption, clinical attempts to muzzle this cell in OA have been frustratingly hit-or-miss. This lack of consistency points to a biological reality we can no longer ignore: the myth of the uniform osteoclast. Patients differ fundamentally—not just in whether osteoclast-mediated pathways drive their specific disease, but in which cellular “program” is at the helm at any given moment [6,50].

**Figure 3:**
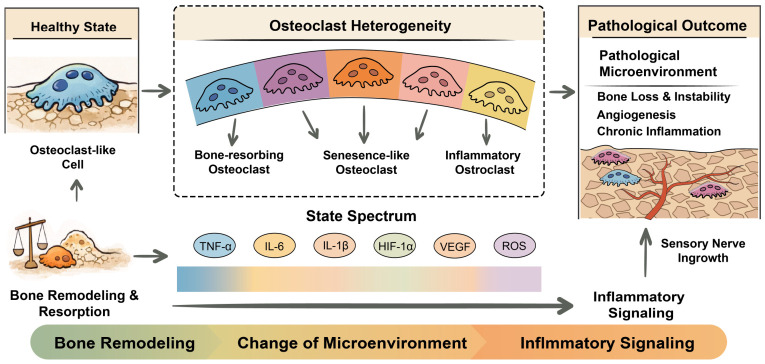
Program-specific translational framework of osteoclast heterogeneity in osteoarthritis. Schematic model linking OA endotypes to dominant osteoclast-lineage programs and corresponding therapeutic opportunities. Rather than treating osteoclast activity as biologically uniform, this framework supports mechanism-matched intervention based on the dominant resorptive, angiogenic-coupling, pain-coupled, or inflammatory/paracrine program operating in a given lesion context.

A clinical framework informed by this diversity demands a more nuanced calculus. We must move beyond the “blunt instrument” of global suppression and recognize that different anti-osteoclast strategies preferentially hit distinct functional programs. Therapeutic success, therefore, hinges on a precise alignment between a drug’s mechanism of action and the operative cellular program—be it resorption, coupling, angiogenesis, or niche activation. Rather than treating the osteoclast as a monolithic entity, we propose that meaningful intervention will only emerge through program-selective therapy [51,52,53]. Ultimately, the pivotal clinical question is no longer *whether* the osteoclast should be targeted in OA, but rather *which* specific lineage-program must be neutralized within a given disease context. This translational shift in logic is summarized in Figure 3.

### 5.1. Osteoclast Heterogeneity for OA Pharmacotherapy

OA trials have often enrolled broadly defined cohorts based primarily on radiographic criteria, although the drivers most relevant to osteoclast biology are clearly heterogeneous. Some patients exhibit bone-active features, such as prominent BMLs, whereas in others the dominant pathology is more closely related to synovitis, meniscal damage, metabolic disturbance, or central sensitization. Under these conditions, the effects of antiresorptive agents are expected to be diluted when such therapies are tested in unselected populations, because any benefit may be confined to subgroups in which bone-driven pathology is dominant [6,50,54]. In early OA, elevated subchondral turnover suggests that suppression of resorptive programs may reduce microdamage propagation and modify BML dynamics. By contrast, in later disease characterized by sclerosis, altered bone quality may reflect maladaptive coupling and niche signaling, including anti-anabolic cues, aberrant angiogenesis, and growth factor activation, all of which may be insufficiently addressed by resorption blockade alone [6,50,52,55,56].

A randomized trial showed that zoledronic acid reduced BML size and knee pain over 12 months, a finding consistent with clinical benefit in the setting of an active high-turnover, osteoclast-driven remodeling program [54]. At the same time, the overall bisphosphonate trial literature remains heterogeneous, suggesting that treatment responsiveness is likely restricted to endotypes in which osteoclast-mediated resorption is a dominant pathogenic process, often in BML-enriched and pain-associated disease [6,50,54,57]. The relevant clinical question is therefore not simply whether bisphosphonates are effective in OA in general, but whether they suppress the operative osteoclast program in a particular patient. Cathepsin K inhibition provides a complementary program-selective strategy by targeting a core osteoclast effector involved in collagen degradation without necessarily eliminating osteoclast-lineage cells; in a placebo-controlled study, MIV-711 improved bone-related structural outcomes and certain cartilage imaging measures [53]. Taken together, these findings support a shift from broadly targeting osteoclasts toward selectively targeting osteoclast programs, because different therapeutic strategies preferentially interfere with different pathogenic outputs, which is the direct clinical implication of osteoclast heterogeneity.

### 5.2. Precision Classification and Stratification of OA Patients

For osteoclast heterogeneity to be translated into clinical benefit, patient management and trial design in OA need to move toward endotype-based selection. A clinically actionable osteoclast-relevant OA endotype may be defined by evidence of active subchondral remodeling, including prominent MRI-detected BMLs, subchondral attrition, and other imaging features indicative of high turnover, often accompanied by rapid structural progression or early dynamic subchondral alteration; bone turnover markers may serve as supportive enrichment tools, although they are insufficient as standalone criteria [6,50,54,57]. Because synovitis provides osteoclastogenic cytokines, including TNF and IL-1 family signals, that can amplify subchondral osteoclast programs, inflammatory-predominant disease may require a combination of anti-inflammatory treatment and program-selective modulation of bone remodeling rather than antiresorptive monotherapy alone [5,58,59]. Stratification should also be stage-sensitive, given that dominant osteoclast programs may shift from early resorption and niche activation to later coupling, angiogenesis, and sclerosis. Accordingly, classification frameworks should incorporate signatures of active remodeling rather than relying exclusively on Kellgren–Lawrence grade [6,50,55].

### 5.3. Personalized, Endotype-Targeted Therapy Based on Osteoclast Heterogeneity

Translating the diversity of the osteoclast lineage into clinical practice necessitates a fundamental pivot: we must move from blanket antiresorptive use toward individualized, program-targeted strategies. In the context of OA, this means tailoring the intervention to the specific pathological driver—whether it be aggressive resorption, impaired coupling, or aberrant neurovascular remodeling.

In patients with “bone-active” OA, characterized by a high BML burden and clear imaging evidence of active remodeling, the priority remains the suppression of traditional resorptive programs. Here, bisphosphonates or Cathepsin K inhibitors serve as rational choices, provided their use is titrated against MRI-based monitoring of BML dynamics [54,57]. Yet, the therapeutic landscape is not limited to mineral erosion. We must also address the “maladaptive coupling” driven by osteoclast-derived mediators like Sema4D, which actively stifle bone formation. Selectively blocking these anti-anabolic signals offers a compelling way to restore subchondral bone quality without the risks associated with globally arresting bone turnover [55,60]. Crucially, we cannot overlook the mononuclear intermediates. These pre-osteoclasts act as secretory hubs, driving the PDGF-BB-dependent angiogenesis and neurovascular invasion that define early-stage pain and progression. Targeting these specific secretory outputs, rather than the mature cell itself, represents a frontier for state-selective inhibition that could decouple structural decay from clinical symptoms [51].

### 5.4. Developing Molecular Targets to Address Osteoclast Heterogeneity

Shifting from broad therapeutic logic to the molecular level, we find that the true potential of targeting osteoclast heterogeneity lies in specific, high-resolution points of intervention. Acknowledging this diversity forces us to abandon the “blunt instrument” of generic anti-resorptives in favor of strategies tailored to the precise program driving a patient’s unique disease endotype.

Several molecular targets currently lead this transition. First is the classic resorptive machinery, where Cathepsin K remains the prototype. However, the goal has evolved toward function-selective inhibition—stopping matrix degradation while leaving vital bone-coupling processes intact [60,61]. Then there are the anti-anabolic signals, such as osteoclast-derived Sema4D; blocking these pathways offers a way to improve the quality of bone remodeling without the risks of global turnover suppression [55]. Perhaps more intriguing are the secretory programs of mononuclear precursors. Pre-osteoclast-derived PDGF-BB, for instance, acts as a critical bridge linking pathological angiogenesis directly to osteochondral decay [51]. Beyond direct signaling, the osteoclast also “licenses” the niche by releasing sequestered growth factors like TGF-β—an aberrant pathway that drives subchondral dysfunction when left unchecked [52]. Finally, upstream osteoimmune signals in inflammatory endotypes provide a robust rationale for combination therapies that address the immune-driven “priming” of osteoclastogenesis [58,59,62]. Collectively, these targets move us away from a one-size-fits-all approach and toward a precision medicine framework for OA.

## 6. Current Controversies and Open Questions

Despite growing support for osteoclast heterogeneity as a useful framework in OA, several important questions remain unresolved. Current single-cell and spatial studies define the osteoclastogenic ecosystem more clearly than the terminal taxonomy of mature osteoclasts in native OA tissue [23,24,25,26,27,28,29]. As a result, some components of the model are supported by relatively direct evidence, whereas others remain inference-based or conceptually provisional. The main open questions concern how completely mature OA osteoclast states have been mapped, whether chondroclasts represent a distinct clastic lineage or an interface-adapted osteoclast state, and to what extent inflammatory osteoclast states and osteomorph recycling have been directly established in OA.

### 6.1. How Completely Have Mature OA Osteoclast States Been Mapped?

Current single-cell and spatial datasets convincingly define the ecosystems that support osteoclast-lineage activation in OA, but they do not yet provide a complete and universally accepted atlas of mature multinucleated osteoclast states in native OA tissue [23,24,25,26,27,28,29]. The best-resolved populations are endothelial, stromal, osteogenic, chondrogenic, and myeloid compartments, whereas mature osteoclasts remain under-captured. It is therefore justified to speak of osteoclast-lineage heterogeneity in OA, but less justified to imply that the terminal taxonomy of mature OA osteoclasts is already settled.

### 6.2. Are Chondroclasts a Distinct Lineage or Interface-Adapted Osteoclast States?

Another important open question concerns the status of chondroclast. Available evidence increasingly supports the view that chondroclasts in OA are not a wholly separate clastic lineage, but rather osteoclast-lineage states adapted to the mineralized cartilage interface [13,43]. This interpretation is more consistent with current concepts of lineage plasticity and local niche instruction, although the degree of divergence from canonical bone-resorbing osteoclasts remains unclear [43]. At present, the most defensible formulation is to regard chondroclasts as interface-adapted osteoclast-lineage states rather than as a definitively distinct cell type.

### 6.3. How Much of Inflammatory Osteoclast Biology and Osteomorph Recycling Is Directly Established in OA?

A similar caution applies to inflammatory osteoclast-lineage states and osteomorph biology. In OA, inflammatory clastic activity is biologically plausible and partly supported by histopathology and niche data, especially in synovitis-associated disease and BML-rich lesions [27,47]. However, much of the mechanistic richness of the “inflammatory osteoclast” concept derives from rheumatoid arthritis and other inflammatory bone-loss settings rather than from direct OA-specific single-cell mapping [13]. Osteomorph biology is also highly relevant conceptually, particularly in fluctuating remodeling environments, but direct evidence for osteomorph function in human OA remains limited [19]. These programs should therefore remain within the OA framework, but with language proportionate to the current strength of the evidence.

## 7. Conclusions

Osteoclast heterogeneity has matured from a mere descriptive curiosity into a functional framework that fundamentally reshapes our understanding of OA. High-resolution spatial and single-cell datasets have effectively dismantled the notion of a monolithic osteoclast lineage. What we see instead is a complex mosaic—multiple cellular states recruited from diverse precursor reservoirs and fine-tuned by the shifting demands of the local, stage-specific niche [13,23,31]. This realization forces a necessary clinical pivot: we can no longer presume the identity of the pathogenic osteoclast axis in a given patient; we must actively identify it [7].

Such mechanical depth finally offers a plausible explanation for why broad “anti-osteoclast” trials have historically struggled with inconsistent outcomes [2,3]. More importantly, it redraws our translational roadmap. The priority is no longer the blunt, indiscriminate suppression of all osteoclasts. Instead, the field must pursue a more surgical, program-selective intervention. The challenge—and the opportunity—lies in disabling the specific program driving an OA endotype while leaving the homeostatic remodeling essential for skeletal health untouched [7,31]. By leveraging lineage-specific multi-omics, we now have the analytical toolkit to link microenvironmental cues directly to patient stratification. This transition is essential for the future of the field: moving OA care away from reactive symptom management and toward a truly mechanism-based disease modification [23,63].

## Data Availability

Data availability is not applicable to this article as no new data were created or analyzed in this study.
